# Observational Follow-up Study on a Cohort of Children with Severe Pneumonia after Discharge from a Day-care Clinic in Dhaka, Bangladesh

**Published:** 2014-06

**Authors:** Hasan Ashraf, Nur H. Alam, Mohammod J. Chisti, Mohammed A. Salam, Tahmeed Ahmed, Niklaus Gyr

**Affiliations:** ^1^Centre for Nutrition and Food Security (CNFS), icddr,b, GPO Box 128, Dhaka 1000, Bangladesh; ^2^Department of Internal Medicine, University of Basel, Switzerland

**Keywords:** Compliance, Day-care, Follow-up, Hospitalization, Morbidity, Mortality, Severe pneumonia, Very severe pneumonia, Bangladesh

## Abstract

Compliance, morbidity, mortality, and hospitalization during fortnightly follow-up were evaluated by an observational study on a cohort of children with severe and very severe pneumonia after day-care treatment at an urban clinic. The primary outcome measures were proportions of success (compliance) and failure (non-compliance) of follow-up visits at the day-care clinic. In total, 251 children were followed up, with median (IQR) age of 5.0 (3.0-9.0) months, and their compliance dropped from 92% at the first to 85% at the sixth visit. Cough (28%), fever (20%), and rapid breathing (13%) were common morbidities. Successful follow-up visits were possible in 180 (95.2%) and 56 (90.3%) of the children with severe and very severe pneumonia respectively. Eleven (4.4%) needed hospitalization, and four (1.6%) died. Majority (≈90%) of the children could be successfully followed up; some failed to attend their scheduled follow-up visits due to hospitalization and death. The common morbidities indicate the importance of follow-up for detecting medical problems and early treatment, thus reducing risk of death.

## INTRODUCTION

Pneumonia is one of the world's leading causes of morbidity and mortality in under-5 children, causing about 1.6 million deaths every year globally (1). According to a report published in 2010, 18% of the total 8.8 million global deaths among under-5 children in 2008 were due to pneumonia (1). There is an estimated 150 million new cases of childhood pneumonia each year globally; conditions of 11 to 20 million of those children with pneumonia were severe enough to be life-threatening, thus requiring hospitalization in developing countries (2-4). In Bangladesh, pneumonia accounts for about 14% and 12% of deaths from community-based (1) and hospital-based study (5) of under-5 children respectively. Some of the death cases in the community might be due to the inadequate access to the hospital facility.

Standard management of severe and very severe pneumonia requires hospitalization for supportive treatment, including oxygen therapy, airway suctioning, fluid and nutritional management, antibiotics, and careful monitoring (6-8). Most, if not all, developing countries (including Bangladesh) do not have enough paediatric beds in hospitals to accommodate the demand for admission of all children with severe and very severe pneumonia. In addition, mothers of ill children have other childcare and household responsibilities that constrain their ability to attend the children during hospitalization, which is often mandatory. Furthermore, transportation, cost, distance from hospital, and lack of adequate childcare are also huge limitations to hospitalization. This inadequate capacity assuredly results in excess and unwanted death of children who, with proper care, would otherwise survive. Alternative treatment options, such as ‘day-care model’ (9-13) are, therefore, needed for those children who cannot be hospitalized but are too sick to be managed in the community through establishment of facilities at ‘outpatient clinics’ for the management of common childhood illnesses, thereby hoping to reduce morbidity and deaths. To address the issue of the day-care management of severe pneumonia, we tested this approach first in an uncontrolled, prospective study (10), followed by another randomized controlled trial (11); the results of both approaches demonstrated that it is possible to manage childhood pneumonia with antibiotics, feeding, and supportive care at the day-care clinics successfully and safely, with efficacy similar to hospital management (10,11). However, there is scarcity of information about what happens to children after recovery from severe/very severe pneumonia and whether this apparent resolution of pneumonia at discharge is sustained in their homes over a long period. Short-term successes, if not sustained, and/or if associated with higher rates of relapses or hospitalizations or deaths, would not be acceptable as a public health intervention. In this communication, we report the compliance, morbidity, mortality, and hospitalization of children with severe/very severe pneumonia over a 3-month period following their successful treatment at day-care clinic.

## MATERIALS AND METHODS

### Objective

The objective of the study was to describe the features of compliance, morbidity, mortality, and hospitalization following discharge from the day-care clinic after successful treatment of severe and very severe pneumonia in children aged <5 years in urban Dhaka, Bangladesh.

### Study design

This is an observational, uncontrolled study following a cohort of children with severe and very severe pneumonia after their discharge from the day-care clinic in an urban setting after recovery from severe or very severe pneumonia. The children were followed up for three months post-management every two weeks (10).

### Setting

The day-care model study (10) and the follow-up study were conducted from 2003 through 2006 at the Radda Clinic, Mirpur, Section 10, Dhaka, Bangladesh, about 5 km from the Dhaka Hospital of icddr,b. The Radda Clinic remained open from 08:00 to 17:00 hours each day, including the weekends and holidays. Adequate space for 12 beds in two separate rooms was established at the Radda Clinic; additional staff was hired for the operation of the clinic every day of the week, which comprised a physician, two nurses, and four healthcare workers to provide care to children from 08:00 to 17:00 hours, and they remained on call beyond these working hours. The vital signs, such as pulse rate, respiration rate, body temperature, weight, and oxygen saturation, were also noted on a daily basis. The health workers were trained to prepare and administer diets to the children and educate and motivate mothers to comply with therapies and follow-up. Provisions for oxygen therapy, nasopharyngeal electric suction therapy, pulse oximeter, and weighing scale were made available at the Radda Clinic (10). All the study children were brought to the clinic on a daily basis at the day-care clinic where they stayed from 08:00 to 17:00 hours and then went back home. Oxygen was administered to all hypoxaemic children; appropriate feeding was given by providing locally-prepared milk-based diet, milk-suji, including breastfeeding for the breastfed ones. Children also received intramuscular injection ceftriaxone administered only once daily in a dose of 75-100 mg/kg body-weight for at least 5 days. The mean (SD) duration of stay at the day-care clinic was 7 (2) days for children with both severe and very severe pneumonia (10). Mothers usually visited the clinic for follow-up during the morning hours from 09:00 to 12:00 but only a few visited in the afternoon. All the project staff, including study physicians, nurses, and healthcare workers, was available at the clinic during the opening hours. Some children with severe/very severe pneumonia were referred from the day-care clinic to the hospital, especially at the closing time of the clinic at 17:00 hours. The included children comprised those with hypoxaemia, severe respiratory distress (respiration rate >70 breaths/minute) and children with associated illnesses, such as ventricular septal defect (VSD), underlying pulmonary tuberculosis, heart failure, etc. (10).

### Participants

In the original reference study (10), we evaluated the day-care treatment of children of either gender, aged 2 to 59 months, with severe or very severe pneumonia according to World Health Organization (WHO) criteria (6-8), without severe acute malnutrition [(SAM, either defined as children having either less than −3 weight-for-height/length z-score −3 ZWH, or bilateral pitting oedema, or mid-upper arm-circumference (MUAC) <115 mm]. They are living within a radius of 5 km from the day-care clinic. The distance of 5 km was taken arbitrarily as they came to the clinic on a daily basis. We provided transportation cost for those children who were very poor and asked for it. From 2003 through 2006, children were enrolled, treated, discharged, and then followed-up for three months at the Radda Clinic.

### Ethics

The original observational study, including the follow-up (10), was approved by the Research Review Committee (RRC) and Ethical Review Committee (ERC) of icddr,b. A written informed consent for participating in the original reference study, including a 3-month follow-up (10) after discharge, was obtained from the parents of each child before enrollment.

### Follow-up study

After successful day-care treatment with antibiotics at the Radda Clinic, feeding, and supportive care during the acute phase, children were discharged (10). Success of day-care treatment was defined as improvement in clinical condition without referral to hospital, full compliance with the day-care treatment until recovery, without premature discontinuation of the study for any reason, and not dying during the study (10). At the time of discharge, the study physician provided all the scheduled dates for follow-up visits for the next three months in the discharge certificate, and parents were asked to visit the clinic every 2 weeks for 3 months. At each of the follow-up visit, the study physician asked the accompanying mothers/caregivers about the health problems of their children since the previous visit. Morbidity data, such as on respiratory illnesses (e.g. cough, fever, runny nose, difficulty in breathing, chest-indrawing, rales on auscultation) and diarrhoeal or other illnesses (e.g. eye, ear, nose, and skin infection, thrush, passage of worms) were noted on a predesigned case report form (CRF). The vital signs, such as pulse rate, respiration rate, body temperature, weight, length/height, MUAC, and oxygen saturation, were also noted at each of the follow-up visits. To assess the changes in nutritional status, the z-scores for weight-for-age (ZWA), weight-for-height (ZWH), and height-for-age (ZHA) were calculated, and all the hospitalizations and deaths were recorded. Any child who developed pneumonia, diarrhoea, or other complications indicating hospitalization during the follow-up period was referred to the Dhaka Hospital of icddr,b or the Institute of Child Health and Shishu Sasthya Foundation Hospital (ICHSH) for necessary treatment. All the events related to hospitalization of each child during the follow-up period were routinely recorded, and minor illnesses were evaluated and treated at the clinic by the study physician. For children who failed to visit the clinic on any given scheduled date of follow-up, health workers in the study team visited their homes next morning, thus encouraging them to comply with the follow-up visits and accompanied them back to the clinic on the same day for recording all necessary information by physicians in the study team.

### Data analysis, including primary outcome measures

Success of follow-up after day-care treatment was defined as the compliance with each of the scheduled follow-up visits for three months every fortnight, either spontaneously or brought by the health workers. However, the failure of follow-up after day-care management really indicated the need for hospitalization or the chance of death anytime during the three months of follow-up period. All data were collected on pre-designed CRFs, edited, entered into a personal computer, and analyzed using Statistical Package for Social Sciences (version 11.5; SPSS Inc., Chicago, Illinois, USA); EPI Info (version 6.0); and CIA. The primary outcome measures were expressed as the proportion of success (compliance) and failure (non-compliance) of follow-up visits after day-care management as determined by estimating odds ratios (ORs) with their 95% confidence intervals (95% CIs). The study groups of severe pneumonia and very severe pneumonia were compared at the time of discharge from the clinic and also during the whole follow-up period. The continuous variables were compared between the two groups, using Student's *t*-test and non-parametric tests. Dichotomous variables were compared using the χ^2^-test or Fisher's exact test, as appropriate. A probability of <0.05 was considered statistically significant.

## RESULTS

Two hundred fifty-one (251) children with severe or very severe pneumonia, residing in Mirpur, Dhaka, attending the day-care clinic and fulfilling the eligibility criteria were enrolled into the original observational study (10). The children were then successfully treated at the clinic and finally followed up for 3 months after discharge. The health status of the study children at the time of discharge from the clinic is shown in Table 1. The median (IQR) age of the children was 5.0 (3.0-9.0) months; 84% were infants (2-11 months), 63% were male, and 91% were breastfed (Table 1). Majority (78%) of the children belonged to poor families with a monthly income of ≈70 US dollar only while 85% of the mothers were housewives, and 71% of the fathers were either day-labourer or rickshaw-puller.

Table 2 shows the compliance, morbidity, and medication received during each of the follow-up visits. The compliance with follow-up visits after discharge from the clinic gradually dropped over time. The common morbidities noted during the follow-up visits were cough (28%), fever (20%), and rapid breathing (13%) while less common symptoms were diarrhoea (7%), difficult breathing (5%), feeding difficulty (4%), and chest wall-indrawing (4%) (Table 2). About 15% of the children suffered from minor illnesses, such as poor appetite, otitis media, conjunctivitis, thrush, scabies, and other skin infections but only 8% of them needed medications during the follow-up period (Table 2), such as syrup amoxicillin, co-trimoxazole, erythromycin, flucloxacillin, chloramphenicol, nalidixic acid, mebendazole, salbutamol, paracetamol as well as tablet iron, folic acid, riboflavin, multivitamin and nystatin drops. Successful follow-up visits for three months were possible in 180/189 (95.2%) children who had severe pneumonia while it was possible in 56/62 (90.3%) children who had very severe pneumonia (Table 3). However, the remaining children who failed to attend the follow-up visits after the day-care treatment were either hospitalized or died (Table 3). Only 11 (4.4%) children needed hospitalization during the follow-up period due to severe pneumonia with hypoxaemia (n=5), ventricular septal defect (VSD) with cyanosis (n=2), pulmonary tuberculosis (n=1), severe pallor (n=1), vesical calculus (n=1), and severe pneumonia with SAM (n=1) (Figure). There were actually no deaths during the acute-phase care (10); however, during the follow-up period, four (1.6%) children died (Table 3) and two of them at the Dhaka Hospital of icddr,b, each due to very severe pneumonia with hypoxaemia and hospital-acquired sepsis (Figure). The causes of death of two other children could not be determined as they died either at home or other healthcare facilities, and the fatality information was not immediately available (Figure).

**Table 1.** Comparison of health status of the study children at the time of discharge

Characteristics	Severe pneumonia (n=189)	Very severe pneumonia (n=62)	Total (n=251)	p value
Male, n (%)	123 (65)	36 (58)	159 (63)	0.32
Age in months, mean (SD)	7.38 (6.52)	6.45 (8.33)	7.15 (7.00)	0.36
Median age, month (IQR)	5.0 (3.0-9.0)	4.0 (3.0-7.25)	5.0 (3.0-9.0)	0.10
Infants (2-11 months), n (%)	157 (83)	55 (89)	212 (84)	0.28
Children (12-59 months), n (%)	32 (17)	7 (11)	39 (16)	0.28
Breastfed, n (%)	170 (90)	58 (93)	228 (91)	0.39
Weight (kg) on discharge, mean (SD)	6.27 (1.79)	5.75 (1.68)	6.14 (1.77)	0.05
Height (cm) on discharge, mean (SD)	63.39 (8.27)	61.11 (7.63)	62.82 (8.16)	0.06
MUAC (cm) on discharge, mean (SD)	12.67 (1.31)	12.33 (1.22)	12.59 (1.29)	0.08
Weight-for-age (%), mean (SD)	83.27 (12.23)	81.13 (14.87)	82.73 (12.94)	0.27
Weight-for-height (%), mean (SD)	97.13 (10.41)	97.63 (9.64)	97.25 (10.21)	0.74
Height-for-age (%), mean (SD)	93.57 (6.92)	92.84 (5.98)	93.39 (6.69)	0.47
Weight-for-age z-score (WAZ), mean (SD)	−1.68 (1.14)	−1.92 (1.43)	−1.74 (1.22)	0.19
Weight-for-height z-score (WHZ), mean (SD)	−0.69 (1.12)	−0.73 (1.14)	−0.70 (1.13)	0.83
Height-for-age z-score (HAZ), mean (SD)	−1.69 (1.14)	−1.92 (1.80)	−1.74 (1.22)	0.23
Difficulty in breathing, n (%)	0	0	0	NA
Lower chest wall-indrawing, n (%)	0	0	0	NA
Hypoxaemia on discharge (oxygen saturation of <95%), n (%)	0	0	0	NA
Length of stay at clinic (days), mean (SD)	7.02 (2.42)	7.11 (2.06)	7.04 (2.34)	0.81
Median length of stay at clinic, days (IQR)	6.0 (6.0-8.0)	7.0 (6.0-8.0)	6.50 (6.0-8.0)	0.24

MUAC=Mid-upper arm-circumference; NA=Not available; SD=Standard deviation; IQR=Interquartile range

**Table 2.** Compliance rates, signs and symptoms, and medication received during each of the follow-up visits

Morbidity/Measure	1st visit	2nd visit	3rd visit	4th visit	5th visit	6th visit	Total visits
Follow-up compliance	231 (92)	234 (93)	228 (91)	219 (87)	213 (85)	212 (85)	1,337 (89)
Cough	71 (28)	78 (31)	68 (27)	73 (29)	67 (27)	58 (23)	415 (28)
Fever	35 (14)	78 (31)	48 (19)	55 (25)	43 (17)	36 (14)	295 (20)
Rapid breathing	35 (14)	37 (14)	39 (15)	26 (10)	23 (9)	31 (12)	191 (13)
Difficult breathing	11 (4)	16 (6)	16 (6)	9 (4)	9 (4)	10 (4)	71 (5)
Feeding difficulty	9 (4)	13 (5)	12 (5)	12 (5)	10 (4)	8 (3)	64 (4)
Chest-indrawing	9 (4)	14 (6)	13 (5)	7 (3)	6 (2)	8 (3)	57 (4)
Diarrhoea	20 (8)	12 (5)	23 (9)	19 (8)	18 (7)	16 (6)	108 (7)
Minor illnesses	30 (12)	45 (18)	44 (17)	37 (15)	32 (13)	34 (13)	222 (15)
Medicine taken	22 (9)	26 (10)	22 (9)	7 (7)	24 (10)	21 (8)	122 (8)

Values are number (%); All the above follow-up visits were done fortnightly; Rapid breathing is defined as age-specific respiration rates of more than 50 breaths per minute for infants aged 2 to 11 months and more than 40 breaths per minute for children aged 12 to 59 months; Lower chest-wall-indrawing was defined as a condition where the lower chest-wall goes inward when the child inhales

**Table 3.** Final outcome for the study children during follow-up period

Outcome	Severe pneumonia (n=189)	Very severe pneumonia (n=62)	Total (n=251)	OR with 95% CI	p value
Successful follow-up visits	180 (95.2%)	56 (90.3%)	236 (94%)	2.14 (0.64-6.96)	0.13
Hospitalization	6 (3.2%)	5 (8.1%)	11 (4.4%)	0.37 (0.10-1.48)	0.10
Death	3 (1.6%)	1 (1.6%)	4 (1.6%)	0.98 (0.09-25.01)	0.68

## DISCUSSION

Majority (≈90%) of the children who suffered from severe and very severe pneumonia can comply with follow-up scheduled fortnightly for 3 months after discharge from the day-care clinic. There was a low incidence (15%) of minor illnesses during the follow-up period, with an even lower requirement of medication (8%) following recovery from severe and very severe pneumonia. It may be mentioned that the illnesses during follow-up period and hospitalization had no relationship with initial pneumonia episode.

Our study showed a great importance of follow-up after discharge from the day-care clinic as some children developed morbidity, which might had chances to lead to a fatal outcome if not documented in time; others needed hospitalization, and a small number died. Our results also indicate the necessity of establishing routine follow-up system for children following successful treatment for severe or very severe pneumonia in healthcare facilities for detecting medical problems early, understanding appropriate intervention and, thus, preventing death. Such follow-ups should be ideally done at the same healthcare facility from where children had received their initial treatment but a community follow-up system may be feasible, if adequately trained and motivated community health workers and other resources are available (12-15).

The follow-up compliance rates were significantly better in the current group of children recovering from severe and very severe pneumonia (10) compared to another group of children recovering from SAM (9,13) in a previous study (OR 1.72; p<0.001). However, a significantly increased number (16%) of children recovering from SAM (9,13) needed medication for various illnesses in contrast to children recovering from severe and very severe pneumonia (8%) during their follow-up period (OR 1.63; p<0.001). Similarly, significantly higher rates of morbidity, such as cough, fever, and diarrhoea were noted in children recovering from SAM (9,13) than those recovering from severe and very severe pneumonia (11,12) (OR 1.30; p<0.01). The higher prevalence of cough, fever, and diarrhoea among children recovering from SAM (9,13) could be explained by their poor nutritional status and the resulting reduced host immunity, making them more vulnerable to the acquisition of new infections during the follow-up period. It might also be explained by the lack of appropriate healthcare knowledge among parents/caregivers of children with SAM, which is an important risk factor for the development of SAM contributed by poor living conditions due to their very low income of about US$ 70/month among most of the parents (72%).

### Limitations

A major limitation of our study is that this was not a randomized controlled trial (RCT) and, thus, the features of follow-up of the day-care children could not be directly compared with those of children with hospital care, which was done in a subsequent RCT (11,12) confirming our study results. Another limitation was the exclusion of children with SAM which is a common underlying condition among children with severe and very severe pneumonia in developing countries and, thus, the features of follow-up of SAM children could not be assessed in this study. We are now conducting another study comparing the efficacy and safety of day care-based management with those of hospital-based management of severe and very severe childhood pneumonia with SAM, including six months of follow-up after discharge, at the Radda Clinic and the ICHSH.

### Conclusions

Successful follow-up visits for three months were possible in more than 90% of the children treated at the day-care clinic, who suffered from severe and very severe pneumonia after discharge from the clinic in spite of some minor failure due to hospitalization and death. Cough, fever, and rapid breathing were the common morbidities experienced during the follow-up period. The finding indicates the importance of follow-up of children with severe and very severe pneumonia at the day-care clinic for the early detection and efficient management of subsequent medical problems, which, in turn, may have an impact in reducing potential future morbidity and death. Our findings highlighted the need for follow-up as part of the overall management of severe and very severe pneumonia and recommendations for an effective community follow-up. The problem of ‘non-compliance with follow-up’ could be addressed through establishment of an effective community follow-up system.

**Figure.**
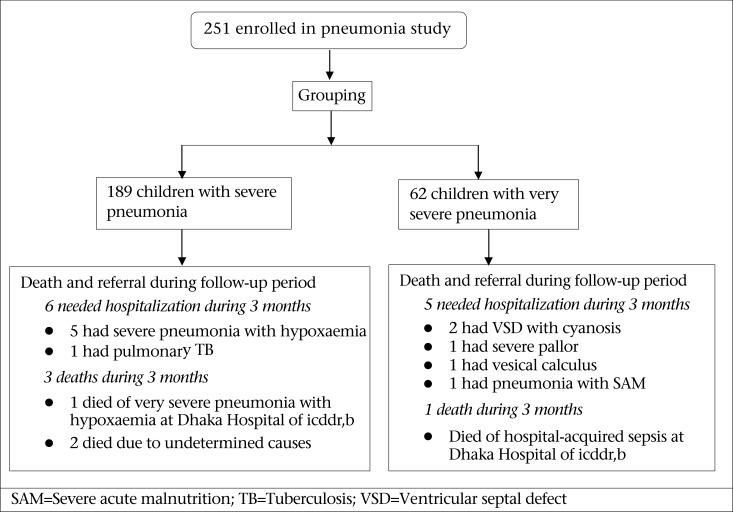
Profile of day-care management of childhood pneumonia with follow-up

## ACKNOWLEDGEMENTS

The study was funded by the Swiss Agency for Development and Cooperation (SDC), Bern; the Gastrointestinal Research Foundation, Liestal; and the University of Basel, Switzerland. icddr,b acknowledges, with gratitude, the commitment of the above donors to its research efforts.
